# Timing of enteral nutrition and clinical outcomes in mild-to-moderate acute pancreatitis: a retrospective cohort study

**DOI:** 10.3389/fnut.2026.1970737

**Published:** 2026-10-29

**Authors:** Dan Song, Wenyuan Song, Peng Li, Hongyan Zhao, Xiaoting Lv

**Affiliations:** 1 Hepatobiliary Surgery, The Second Affiliated Hospital of Hainan Medical University, Haikou, China; 2 Department of Pathology, The Second Affiliated Hospital of Hainan Medical University, Haikou, China

**Keywords:** acute pancreatitis, clinical outcomes, delayed EN, early EN, enteral nutrition, feeding intolerance, hospital stay, nutritional support

## Abstract

**Background:**

The optimal timing of enteral nutrition (EN) in acute pancreatitis (AP) remains clinically important.

**Objective:**

This study evaluated whether early EN improved outcomes compared with delayed EN.

**Methodology:**

This retrospective cohort study included 650 adults admitted with AP from January 2022 through December 2025. Patients were classified into an early EN group (initiation within 48 h; *n* = 392) or a delayed EN group (initiation after 48 h; *n* = 258). Demographic and clinical information, nutritional data, biochemical data, and outcome data were collected from medical records. Multiple regression analysis was used, adjusting for relevant confounders.

**Results:**

Higher nutritional risk, lower albumin, and higher inflammatory burden at admission were all associated with delayed EN. Early EN was associated with shorter hospital stay (5.5 vs. 7.4 days; *p* < 0.001), quicker pain resolution, earlier adequate enteral intake (2.4 vs. 3.5 days; *p* < 0.001), and lower feeding intolerance (8.7% vs. 18.6%; *p* < 0.001). The early EN group also had lower rates of local and systemic complications and ICU transfer. Early EN remained independently associated with lower odds of feeding intolerance after adjustment (OR 0.52, 95% CI: 0.32–0.83; *p* = 0.006), decreased hospital stay (1.21 days, 95% CI: −1.58 to −0.84; *p* < 0.001), recurrent/worsening pain, systemic complications, and ICU transfer after adjustment.

**Conclusion:**

In mild to moderate AP, early EN (within 48 h) was independently associated with more rapid recovery and fewer complications, supporting its use when clinically tolerated.

## 1 Introduction

Acute pancreatitis (AP) is a frequent gastrointestinal disorder with a highly variable clinical course. The Revised Atlanta Classification categorizes AP as mild, moderately severe, or severe. Mild AP is characterized by the absence of organ failure and local or systemic complications; moderately severe AP involves transient organ failure lasting ≤48 h and/or local or systemic complications without persistent organ failure; and severe AP is characterized by persistent organ failure lasting >48 h (1). Nutritional support is a key part of AP management. Historically, clinicians avoided feeding patients with AP because they believed oral feeding would stimulate pancreatic secretion and worsen pancreatic inflammation. This has changed significantly, and early feeding (oral or enteral), if tolerated, is now preferred. Early feeding is linked to shorter hospital stays, better feeding tolerance, and faster clinical recovery (2). Thus, initiating nutrition has become a crucial part of AP management.

Disease severity, systemic inflammatory response, etiology, nutritional status, and gastrointestinal function and tolerance are several patient- and disease-related factors that influence the initiation of enteral nutrition (EN). In mild to moderate AP, gastrointestinal function does not change significantly, allowing early EN (3). Delayed EN may prolong inadequate nutritional intake, particularly when gastrointestinal symptoms interfere with feeding. Clinical tolerance remains important when advancing nutritional intake because gastrointestinal symptoms and feeding intolerance can limit successful oral or enteral feeding in some patients with AP. Although early nutritional support is increasingly recommended, the optimal timing of EN may still depend on disease severity, gastrointestinal tolerance, nutritional status, and the patient’s overall clinical condition.

The association between EN timing and clinical outcomes may involve several physiological mechanisms. AP increases metabolic demand and is linked to gastrointestinal dysfunction, reduced intestinal barrier integrity, and altered gut microbiota (4). EN may maintain gut mucosal integrity and gastrointestinal function and may reduce bacterial translocation and infectious complications, which may improve nutritional status during the inflammatory phase of AP, while prolonged fasting may exacerbate malnutrition (5). These are physiological mechanisms that offer a justification for not prolonging the fast and promoting EN at a suitable time. Observational evidence, however, cannot determine whether earlier EN improves clinical outcomes because nutrition timing may also reflect underlying disease severity and gastrointestinal tolerance (6).

Growing evidence supports the benefits of early nutritional support in AP; however, it remains unclear whether EN timing is linked to clinical outcomes in mild to moderately severe AP. Previous studies have included groups with varying severities, often emphasizing oral refeeding, and used different definitions of early and delayed nutrition (7). More real-world data are therefore needed to further clarify the relationship between EN timing, nutritional tolerance, and clinical recovery. Thus, this retrospective cohort study compared time to EN initiation, feeding tolerance, clinical recovery, inflammatory response, complications, and other in-hospital outcomes between early and delayed EN initiation in patients with mild-to-moderately severe AP.

## 2 Methodology

### 2.1 Study design and setting

This retrospective cohort study compared clinical outcomes between adult patients with mild-to-moderate AP who started EN early and those who started EN later (delayed EN). Medical records of all consecutive eligible patients admitted between January 1, 2022 and December 31, 2025 were collected from the electronic medical records (EMR) and nutrition-management system (NMS) of a tertiary care hospital. The study examined feeding schedules, nutritional tolerance, inflammatory response, hospital stay, and clinical complications.

The Strengthening the Reporting of Observational Studies in Epidemiology (STROBE) checklist for cohort studies was used to report the study. Because the study relied on routinely collected electronic medical record data, the report also followed the Reporting of Studies Conducted Using Observational Routinely Collected Health Data (RECORD) statement. Supplementary materials are included that contain completed STROBE and RECORD checklists.

### 2.2 Study population and eligibility criteria

Adults (aged ≥18 years) who had been diagnosed with mild or moderately severe AP were eligible. AP was diagnosed based on 3 criteria per the Revised Atlanta Classification (1): characteristic abdominal pain, serum amylase/lipase levels more than 3 times the upper reference limit, and imaging findings compatible with AP. Patients were excluded from the study if they had ongoing organ failure/severe AP, chronic pancreatitis, pancreatic malignancy, previous major pancreatic surgery, age <18 years, incomplete nutrition-timing documentation, or inadequate clinical or outcome data.

### 2.3 Patient screening and cohort selection

The hospital admission and diagnostic databases were used to identify potential admissions for AP from January 1, 2022 to December 31, 2025. There were initially 1,086 potentially relevant admission records. Removal of duplicate or repeated records (when each patient has multiple acute episodes) left 990 unique patient records to be eligible for screening. 148 records were excluded at the first screening level as the diagnosis did not meet the criteria for AP and/or there was insufficient documentation to confirm the diagnosis. The other 842 patients were comprehensively assessed using their full medical records.

192 patients were excluded from full medical-record review based on the predefined exclusion criteria: 31 patients aged <18 years, 48 patients with severe AP or persistent organ failure, 27 patients with chronic pancreatic disease or pancreatic malignancy, 14 patients with a history of previous major pancreatic surgery, 39 patients with insufficient documentation of the timing of nutrition initiation, and 33 patients with insufficient baseline or outcome information to support the predefined analyses. So, 650 patients met all the inclusion criteria and formed the final analytic cohort.

In the final cohort, 392 (60.3%) started enteral nutrition within 48 h of admission and were grouped as the early EN group; 258 (39.7%) started enteral nutrition after 48 h of admission and were classified as the delayed EN group. The whole screening and cohort-selection process is shown in Figure 1.

**Figure 1:**
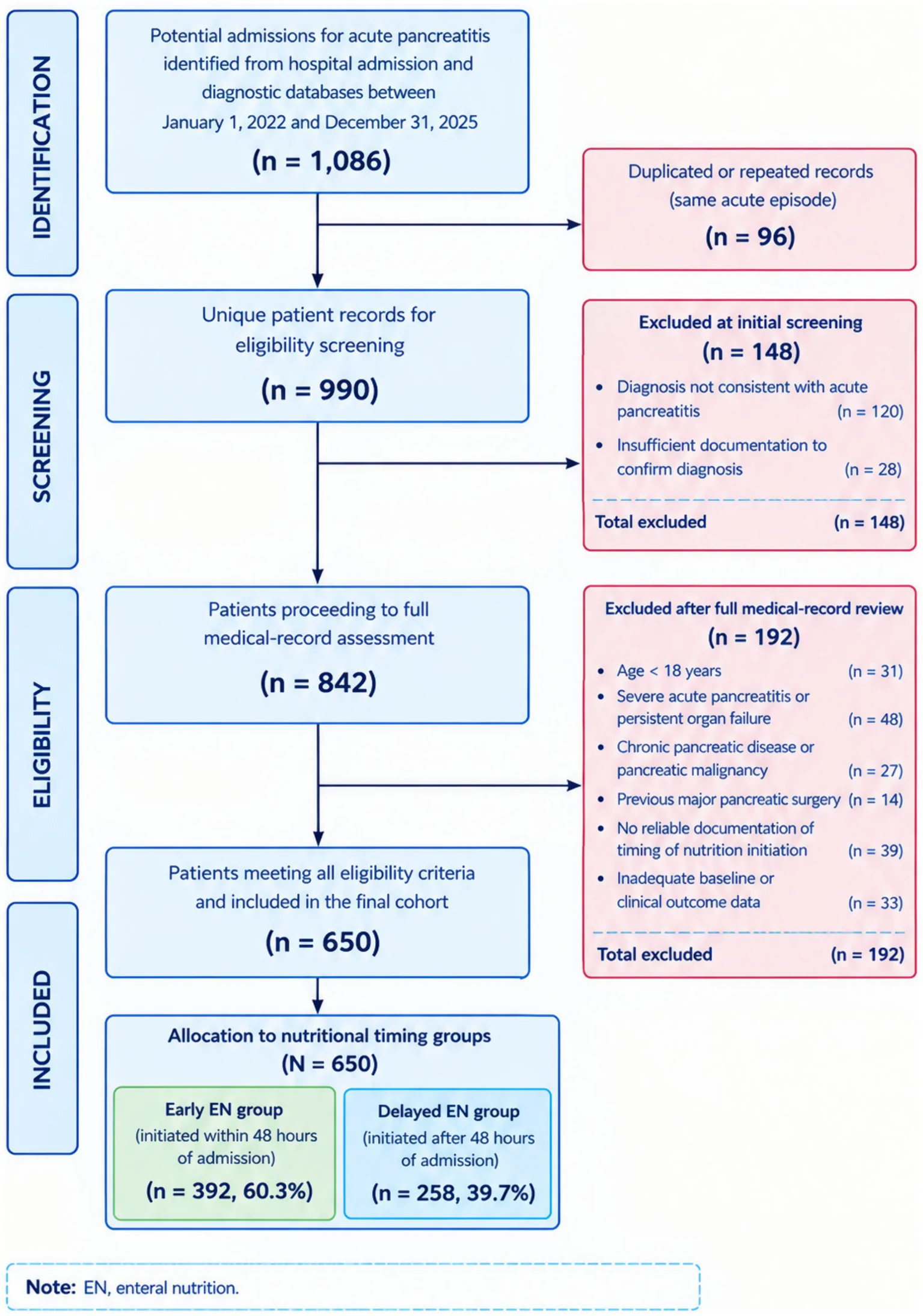
Selection flow diagram of participants.

### 2.4 Assessment of enteral nutrition timing

The main exposure was the time between hospital admission and the start of enteral nutrition. Oral nutritional intake and tube-based enteral feeding were documented as the therapeutic nutritional strategy (enteral nutrition). Patients were mainly divided into two groups: early EN (≤ 48 h) and delayed EN (> 48 h), in accordance with the literature on early EN (8). The time from admission to nutritional initiation was also kept as a continuous variable for secondary and sensitivity analyses. The medical record was reviewed for the following reasons for delayed EN: persisting abdominal pain, nausea/vomiting, ileus, clinical instability, and feeding intolerance.

### 2.5 Clinical, nutritional and laboratory variables

Demographic factors were age, sex, BMI, smoking history and alcohol use. Clinical variables included AP etiology, symptom duration, comorbidities, admission vitals, abdominal pain, nausea/vomiting, and AP severity indicators. Nutritional parameters included BMI, serum albumin, total protein, hemoglobin, lymphocyte count, and Nutritional Risk Screening 2002 (NRS-2002) score (9). Laboratory parameters were amylase, lipase, glucose, CRP, complete blood count, liver enzymes, bilirubin, creatinine, blood urea nitrogen, calcium, triglycerides and electrolytes. The variables were chosen because they may reflect nutritional timing or clinical recovery.

### 2.6 Clinical outcomes

The primary outcome was length of hospital stay, defined as the time from hospital admission to discharge. Secondary clinical outcomes included time to resolution of abdominal pain, time to achievement of adequate enteral/oral intake, feeding intolerance, recurrent or aggravated abdominal pain, progression to moderately severe disease, local and/or systemic complications, transfer to the intensive care unit, invasive intervention, peak C-reactive protein (CRP), CRP reduction by discharge, 30-day readmission, and in-hospital mortality. Nutrition-related outcomes included achievement of adequate intake within the first 72 h, nutritional supplementation, feeding interruptions, and refeeding after interruption.

### 2.7 Operational definitions

Early EN was defined as initiation of oral or enteral tube feeding within 48 h of hospital admission, and “delayed enteral nutrition” was defined as initiation after 48 h (8). Feeding intolerance was defined as any documented nausea, vomiting, abdominal distention, increasing abdominal pain, ileus, or other gastrointestinal symptoms that prevented, reduced, or required modification of the prescribed oral or enteral nutritional intake. Recurrent or worsening abdominal pain was assessed separately as a clinical outcome and referred to recurrence or aggravation of abdominal pain during hospitalization, irrespective of whether it resulted in feeding intolerance.

Adequate enteral/oral intake was defined as meeting 75% of estimated daily energy and protein requirements via EN, with documented tolerance of the prescribed nutrition. The first day on which the patient achieved this level was recorded as the day to adequate intake. Adequate intake within the first 72 h was recorded as a binary outcome.

Nutritional supplementation was the need for extra nutritional support if oral and/or enteral intake was less than 75% of estimated energy and protein needs (10). Supplementation: provided prescribed oral nutritional supplements and/or extra enteral nutrition support in addition to the patient’s usual oral diet. Dietitian/nutritionist assessment, dietetic records, physician and nursing documentation, and electronic nutrition orders in the medical record were used to determine the need for supplementation.

The presence of regular alcohol consumption at baseline (as documented in the medical record) was used to identify regular alcohol exposure. Frequencies and amounts of alcohol use were not always reported on the retrospective records, so alcohol exposure was examined as a binary variable (regular alcohol exposure vs. no regular alcohol exposure).

Feeding interruption was defined as a documented temporary cessation of oral or enteral nutrition after feeding had been initiated, due to gastrointestinal symptoms, deterioration or a clinician’s order to fast. Refeeding after interruption was defined as documented resumption of oral or enteral nutrition following such a temporary interruption.

Invasive intervention was defined as invasive procedure performed during the index hospitalization for the treatment of AP or an AP-related complication. Examples of procedures recorded in the medical records were used to identify this outcome; however, the analysis treated invasive intervention as a binary variable based on the electronic medical record documentation.

The inflammatory response was evaluated through blood C-reactive protein (CRP) levels during the hospital stay. Peak CRP was defined as the highest recorded CRP value during the hospital stay. CRP reduction due to discharge was determined as the percentage reduction of the admission CRP concentration from the discharge concentration.

AP severity was assessed based on the Revised Atlanta Classification (1). Mild AP was defined as AP without organ failure or local or systemic complications, whereas moderately severe AP was defined by transient organ failure lasting ≤48 h and/or local or systemic complications without persistent organ failure.

Abdominal pain intensity was assessed at admission and categorized as mild/none or moderate/severe according to the documented clinical assessment in the medical record.

Hospital stay was defined as the number of days between hospital admission to hospital discharge. Thirty-day readmission was defined as an unplanned hospital readmission within 30 days after discharge from the index hospitalization. In-hospital mortality was defined as death occurring during the index hospitalization.

### 2.8 Data collection and quality assurance

Information was obtained through a standardized data-collection form including demographics, disease descriptors, nutritional exposure details, laboratory parameters, complications, and outcomes. Feeding times were verified with physician notes, nursing documentation, dietary records and electronic nutrition orders, if available. Two investigators independently reviewed a sample of records for eligibility, exposure classification and outcome extraction. The analytical dataset was stripped of identifying information and discrepancies were resolved by consensus or by having a senior investigator review the data.

The EMR was checked for documented meal intake, nutrition assessments by a dietitian/nutritionist, nutritional prescriptions, oral nutritional supplements, enteral feeding orders, feeding interruptions, and documentation by the clinician of tolerance, for nutritional outcomes. If more than one record was present, the documented intake and nutrition orders were compared and the earliest documentation, in which the predefined ≥75% nutritional-intake criterion was documented, was used for the time-to-adequate-intake outcome.

During data extraction and database quality checking, the completeness of all the variables used in the primary and multivariable analyses were checked. No missing values were found for the variables used for the final analyses after baseline or outcome data were missing. Therefore, no statistical imputation was performed, and all 650 patients in the final analytic cohort were included in the respective analyses.

### 2.9 Statistical analysis

Data were analyzed using IBM SPSS Statistics version 29.0. No missing values were identified for variables included in the final analyses; therefore, no statistical imputation was performed. The Shapiro–Wilk test was used to assess the normality of continuous variables, and they were summarized as mean ± SD or median (IQR), as appropriate. Categorical variables were presented as frequencies and percentages. The differences between the nutrition groups (early versus delayed) were analyzed by an independent-samples t test or a Mann–Whitney U test for continuous variables and by a χ^2^ test or a Fisher’s exact test for categorical variables.

Prespecified multivariable regression analyses were used to examine the independent association of enteral nutrition timing with clinical outcomes. The adjusted models included the following 12 variables: age, sex, BMI, severity of AP, etiology of AP, symptom duration, diabetes mellitus, baseline C-reactive protein (CRP), serum albumin, serum creatinine, Nutritional Risk Screening 2002 (NRS-2002) score, nausea/vomiting. *A priori* these were chosen because they held clinical significance as potential determinants of nutrition timing and clinical recovery, and were applied to all models adjusted for these variables.

Multivariable logistic regression was used for binary outcomes and multivariable linear regression was used for continuous outcomes. A sensitivity analysis was performed by modeling the exposure as time since admission and log-transforming the exposure (time since admission/12 h). Graphical presentation of the continuous exposure analysis involved plotting patient-level observations on the x-axis (days from admission to nutrition initiation) and y-axis (days of hospital stay). The analytic cohort included 271 patients, with one patient per point. No bining or aggregation of observations were done. The overall association and uncertainty was represented by a fitted linear regression line with a 95% confidence interval band.

The multivariable estimates of in-hospital mortality, invasive intervention and transfer to ICU were deemed exploratory due to the low number of events for each outcome. To obtain adjusted estimates, these models were kept in place, but they should be interpreted with caution due to possible overfitting and potentially imprecise or unstable estimates. So, these results were not regarded as conclusive evidence of independent associations. Variance inflation factors were used to assess multicollinearity. Reported effect estimates are adjusted estimates with 95% confidence intervals. The *p*-value was calculated on both sides and was considered to be statistically significant when *p* < 0.05. Because multiple secondary clinical outcomes were evaluated, no formal multiplicity adjustment was applied; therefore, findings for secondary outcomes were interpreted as exploratory, and the reported *p*-values were not considered confirmatory evidence of independent effects.

To reduce potential sources of bias, predefined selection criteria were used, definitions of severity of AP were clearly established and of nutritional exposure, information on key data were missing, records with insufficient key data were excluded, clinically relevant confounding factors were accounted for in multiple regression models, and sensitivity analyses were conducted by using nutritional exposure as a continuous variable.

### 2.10 Ethical considerations

Before data collection, the relevant Ethics Committee approved the study protocol. This research was approved by the Ethics Committee of The Second Affiliated Hospital of Hainan Medical University, Approval Number: NO. LC20251215. Because this was a retrospective study of previously recorded clinical data and there was no change in patient management, informed consent was not required per institutional guidelines. The study followed the principles and standards of the Declaration of Helsinki and institutional and national ethical standards. Patient confidentiality was observed at all stages of data extraction, analysis, and reporting, and all identifying data were removed from the dataset used for the study.

## 3 Results

### 3.1 Baseline demographic and clinical characteristics

The baseline demographic and clinical characteristics of the 650 patients are summarized in Table 1. The cohort’s mean age was 49.6 ± 14.7 years, and 361 patients (55.5%) were male. Biliary disease was the predominant etiology, accounting for 359 patients (55.2%), followed by hypertriglyceridemia in 121 (18.6%). Overall, 521 patients (80.2%) had mild AP and 129 (19.8%) had moderately severe AP. Although most demographic characteristics were comparable between groups, moderately severe AP was more frequent in the delayed group than in the early group (24.8% vs. 16.6%, *p* = 0.01). Nausea/vomiting at admission was more common among patients receiving delayed EN than in the early EN group (68.2% vs. 52.8%, *p* < 0.001). Moderate-to-severe abdominal pain at admission was present in 71.7% of patients in the delayed-nutrition group compared with 60.2% in the early-nutrition group (*p* = 0.003). All variables included in the primary and multivariable analyses were complete for the 650 patients in the final analytic cohort. No observations were excluded from the final analyses because of missing values, and no statistical imputation was required. Although some vital signs differed significantly between groups at admission, these differences were small and unlikely to be clinically significant for blood pressure and temperature. The results were therefore interpreted in the context of their statistical significance rather than as evidence of substantial baseline physiological differences.

**Table 1:** Baseline demographic and clinical characteristics according to enteral nutrition timing.

Variable	Overall (*N* = 650)	Early ≤48 h (*n* = 392)	Delayed >48 h (*n* = 258)	*p* value
Age, years	49.6 ± 14.7	48.8 ± 14.2	50.8 ± 15.4	0.09
Male sex	361 (55.5)	222 (56.6)	139 (53.9)	0.51
BMI, kg/m^2^	25.1 ± 3.8	24.9 ± 3.7	25.4 ± 3.9	0.11
BMI < 18.5 kg/m^2^	36 (5.5)	18 (4.6)	18 (7.0)	0.21
BMI 18.5–24.9 kg/m^2^	335 (51.5)	206 (52.6)	129 (50.0)	0.53
BMI 25.0–29.9 kg/m^2^	224 (34.5)	138 (35.2)	86 (33.3)	0.64
BMI ≥ 30 kg/m^2^	55 (8.5)	30 (7.7)	25 (9.7)	0.38
Current smoking	143 (22.0)	80 (20.4)	63 (24.4)	0.25
Regular alcohol exposure	118 (18.2)	64 (16.3)	54 (20.9)	0.16
Hypertension	147 (22.6)	80 (20.4)	67 (26.0)	0.11
Type 2 diabetes	93 (14.3)	49 (12.5)	44 (17.1)	0.11
Dyslipidemia	129 (19.8)	70 (17.9)	59 (22.9)	0.14
Chronic kidney disease	32 (4.9)	15 (3.8)	17 (6.6)	0.12
Symptom duration, h	18 (10–31)	17 (9–29)	20 (11–34)	0.03
Admission systolic BP, mmHg	128 ± 17	126 ± 16	131 ± 18	<0.001
Admission heart rate, beats/min	91 ± 14	89 ± 13	94 ± 15	<0.001
Temperature, °C	37.1 ± 0.6	37.0 ± 0.5	37.2 ± 0.6	0.002
Abdominal pain at admission	638 (98.2)	383 (97.7)	255 (98.8)	0.36
Moderate/severe pain intensity	421 (64.8)	236 (60.2)	185 (71.7)	0.003
Nausea/vomiting	383 (58.9)	207 (52.8)	176 (68.2)	<0.001
Abdominal distension	94 (14.5)	45 (11.5)	49 (19.0)	0.01
Mild AP	521 (80.2)	327 (83.4)	194 (75.2)	0.01
Moderately severe AP	129 (19.8)	65 (16.6)	64 (24.8)	0.01
Biliary etiology	359 (55.2)	217 (55.4)	142 (55.0)	0.94
Hypertriglyceridemia	121 (18.6)	76 (19.4)	45 (17.4)	0.54
Alcohol-related	93 (14.3)	55 (14.0)	38 (14.7)	0.81
Other/idiopathic	77 (11.8)	44 (11.2)	33 (12.8)	0.56

Values are presented as mean ± SD, median (IQR), or n (%), as appropriate. All demographic and clinical characteristics presented in Table 1 were assessed at admission unless otherwise indicated. Regular alcohol exposure indicates documented regular alcohol consumption at baseline; frequency and quantity were not consistently available and were not separately analyzed. AP, acute pancreatitis; BMI, body mass index; BP, blood pressure.

### 3.2 Baseline nutritional status, biochemical profile, and inflammatory characteristics

Table 2 presents the nutritional, biochemical, and inflammatory profiles at admission. Patients in the delayed-nutrition group demonstrated a less favorable nutritional and inflammatory profile. The mean serum albumin concentration was 3.61 ± 0.54 g/dL in the delayed group compared with 3.79 ± 0.48 g/dL in the early group (*p* < 0.001), while nutritional risk defined by NRS-2002 ≥ 3 was present in 31.8% versus 20.9%, respectively (*p* = 0.002). Median CRP was also higher among delayed EN patients (65 vs. 45 mg/L, *p* < 0.001). Similarly, leukocyte count, BUN, creatinine, and glucose were modestly higher in the delayed group, suggesting greater systemic inflammatory and metabolic disturbance at admission.

**Table 2:** Baseline nutritional risk, laboratory parameters, and inflammatory profile according to enteral nutrition timing.

Variable	Overall	Early ≤48 h	Delayed >48 h	*p* value
NRS-2002 score	2.1 ± 1.1	1.9 ± 1.0	2.4 ± 1.2	<0.001
NRS-2002 ≥ 3	164 (25.2)	82 (20.9)	82 (31.8)	0.002
Serum albumin, g/dL	3.72 ± 0.51	3.79 ± 0.48	3.61 ± 0.54	<0.001
Hypoalbuminemia	176 (27.1)	91 (23.2)	85 (32.9)	0.007
Total protein, g/dL	6.71 ± 0.66	6.77 ± 0.63	6.62 ± 0.69	0.006
Hemoglobin, g/dL	13.5 ± 1.7	13.6 ± 1.6	13.4 ± 1.8	0.18
Lymphocyte count, ×10^9^/L	1.72 ± 0.62	1.77 ± 0.60	1.65 ± 0.64	0.02
Leukocyte count, ×10^9^/L	11.2 ± 3.6	10.8 ± 3.3	11.8 ± 3.9	<0.001
CRP, mg/L	52 (24–108)	45 (21–91)	65 (29–129)	<0.001
CRP > 100 mg/L	157 (24.2)	76 (19.4)	81 (31.4)	0.001
Amylase, U/L	786 (468–1,426)	759 (451–1,370)	827 (489–1,507)	0.18
Lipase, U/L	1,164 (671–2,146)	1,109 (648–2,034)	1,248 (706–2,310)	0.09
Glucose, mg/dL	132 ± 39	128 ± 36	138 ± 42	0.002
Creatinine, mg/dL	0.92 ± 0.24	0.89 ± 0.21	0.96 ± 0.27	<0.001
BUN, mg/dL	20.8 ± 7.5	19.7 ± 6.9	22.5 ± 8.1	<0.001
Calcium, mg/dL	8.67 ± 0.48	8.71 ± 0.45	8.60 ± 0.52	0.006
Triglycerides, mg/dL	286 (172–421)	278 (166–408)	299 (180–443)	0.17
ALT, U/L	86 (49–157)	82 (46–151)	92 (52–165)	0.12
AST, U/L	71 (39–131)	68 (37–124)	76 (42–142)	0.16
Total bilirubin, mg/dL	1.4 (0.8–2.3)	1.3 (0.8–2.2)	1.5 (0.9–2.5)	0.08
Hypocalcemia	74 (11.4)	36 (9.2)	38 (14.7)	0.03

Values are presented as mean ± SD, median (IQR), or *n* (%). All nutritional and laboratory parameters presented in this table were assessed at admission, before initiation of EN. CRP, C-reactive protein; BUN, blood urea nitrogen; NRS-2002, Nutritional Risk Screening 2002; ALT, alanine aminotransferase; AST, aspartate aminotransferase.

### 3.3 Timing, route, and tolerance of enteral nutrition

The characteristics of nutritional initiation are shown in Table 3. The median time from admission to initiation of nutrition was 31 h (IQR 20–39) in the early group compared with 70 h (IQR 58–91) in the delayed group (*p* < 0.001). Oral nutrition was the initial route in 92.3% of early-fed patients compared with 79.1% of delayed EN patients. In contrast, tube-based enteral nutrition was more frequent in the delayed group (20.9% vs. 7.7%, *p* < 0.001). Feeding interruption occurred in 22.9% of delayed EN patients compared with 9.7% of early-fed patients (*p* < 0.001). Persistent nausea/vomiting, ileus, and abdominal pain were also substantially more frequent reasons for delayed nutritional initiation.

**Table 3:** Timing, route of delivery, and tolerance of enteral nutrition.

Variable	Overall (*N* = 650)	Early ≤48 h	Delayed >48 h	*p* value
Time to nutrition initiation, h	43 (25–67)	31 (20–39)	70 (58–91)	<0.001
Nutrition initiated ≤24 h	189 (29.1)	189 (48.2)	0	<0.001
Nutrition initiated 25–48 h	203 (31.2)	203 (51.8)	0	<0.001
Nutrition initiated 49–72 h	147 (22.6)	0	147 (57.0)	<0.001
Nutrition initiated >72 h	111 (17.1)	0	111 (43.0)	<0.001
Oral nutrition initially	566 (87.1)	362 (92.3)	204 (79.1)	<0.001
Tube-based EN initially	84 (12.9)	30 (7.7)	54 (20.9)	<0.001
Clear/liquid diet initially	235 (36.2)	137 (34.9)	98 (38.0)	0.42
Soft/solid low-fat diet initially	331 (50.9)	225 (57.4)	106 (41.1)	<0.001
Nutritional supplementation required	84 (12.9)	38 (9.7)	46 (17.8)	0.004
Feeding interruption	97 (14.9)	38 (9.7)	59 (22.9)	<0.001
Refeeding after interruption	68 (10.5)	25 (6.4)	43 (16.7)	<0.001
Persistent nausea/vomiting delaying feeding	112 (17.2)	30 (7.7)	82 (31.8)	<0.001
Ileus/gastrointestinal dysfunction	56 (8.6)	16 (4.1)	40 (15.5)	<0.001
Persistent abdominal pain delaying feeding	87 (13.4)	19 (4.8)	68 (26.4)	<0.001
Clinician-directed fasting	104 (16.0)	24 (6.1)	80 (31.0)	<0.001
Adequate enteral/ oral intake achieved within first 72 h	514 (79.1)	346 (88.3)	168 (65.1)	<0.001

Values are presented as median (IQR) or *n* (%). EN, enteral nutrition. Early EN was defined as initiation ≤48 h after admission and delayed EN as initiation >48 h. Estimated daily energy and protein requirements were used to determine adequate enteral/oral intake, defined as achievement of ≥75% of EAR. Adequate enteral/ Oral intake of less than 75% of estimated requirements and nutritional support prescribed was deemed to require nutritional supplementation. Dietary information and nutrition supplements were determined by dietary records, nutrition/dietitian assessment, physician documentation, nursing documentation, and electronic nutrition orders.

### 3.4 Clinical recovery and hospital outcomes

Table 4 presents clinical recovery and hospitalization outcomes by nutritional timing. Patients receiving early EN had a shorter median hospital stay than delayed EN (5.5 vs. 7.4 days, *p* < 0.001). The early group also achieved pain resolution more quickly (2.0 vs. 2.7 days, *P* < 0.001) and reached adequate enteral intake earlier (2.4 vs. 3.5 days, *p* < 0.001). Feeding intolerance occurred in 8.7% of early-fed patients compared with 18.6% of delayed EN patients (*p* < 0.001). Local and systemic complications, ICU transfer, and invasive interventions were numerically less frequent in the early group, whereas in-hospital mortality and 30-day readmission did not differ significantly between groups. The major differences in clinical outcomes according to nutritional timing are summarized graphically in Figure 2.

**Table 4:** Clinical recovery, nutritional tolerance, inflammatory response, and hospitalization outcomes according to enteral nutrition timing.

Outcome	Overall	Early ≤48 h	Delayed >48 h	*p* value
Hospital stay, days	6.2 (4.8–8.4)	5.5 (4.2–7.1)	7.4 (5.7–9.8)	<0.001
Pain resolution, days	2.3 (1.5–3.2)	2.0 (1.3–2.8)	2.7 (1.8–3.7)	<0.001
Time to achievement of adequate enteral/oral intake, days	2.8 (2.0–4.0)	2.4 (1.7–3.4)	3.5 (2.5–4.7)	<0.001
Feeding intolerance	82 (12.6)	34 (8.7)	48 (18.6)	<0.001
Recurrent/worsening pain	103 (15.8)	48 (12.2)	55 (21.3)	0.003
Peak CRP, mg/L	104 (51–194)	91 (47–176)	126 (61–224)	<0.001
CRP reduction by discharge, %	59.3 ± 21.0	63.4 ± 19.2	53.1 ± 22.5	<0.001
Albumin at discharge, g/dL	3.46 ± 0.51	3.55 ± 0.47	3.32 ± 0.52	<0.001
Local complications	57 (8.8)	27 (6.9)	30 (11.6)	0.04
Systemic complications	49 (7.5)	22 (5.6)	27 (10.5)	0.03
ICU transfer	34 (5.2)	13 (3.3)	21 (8.1)	0.01
Invasive intervention	26 (4.0)	10 (2.6)	16 (6.2)	0.03
In-hospital mortality	8 (1.2)	3 (0.8)	5 (1.9)	0.18
30-day readmission	42 (6.5)	20 (5.1)	22 (8.5)	0.09

**Figure 2:**
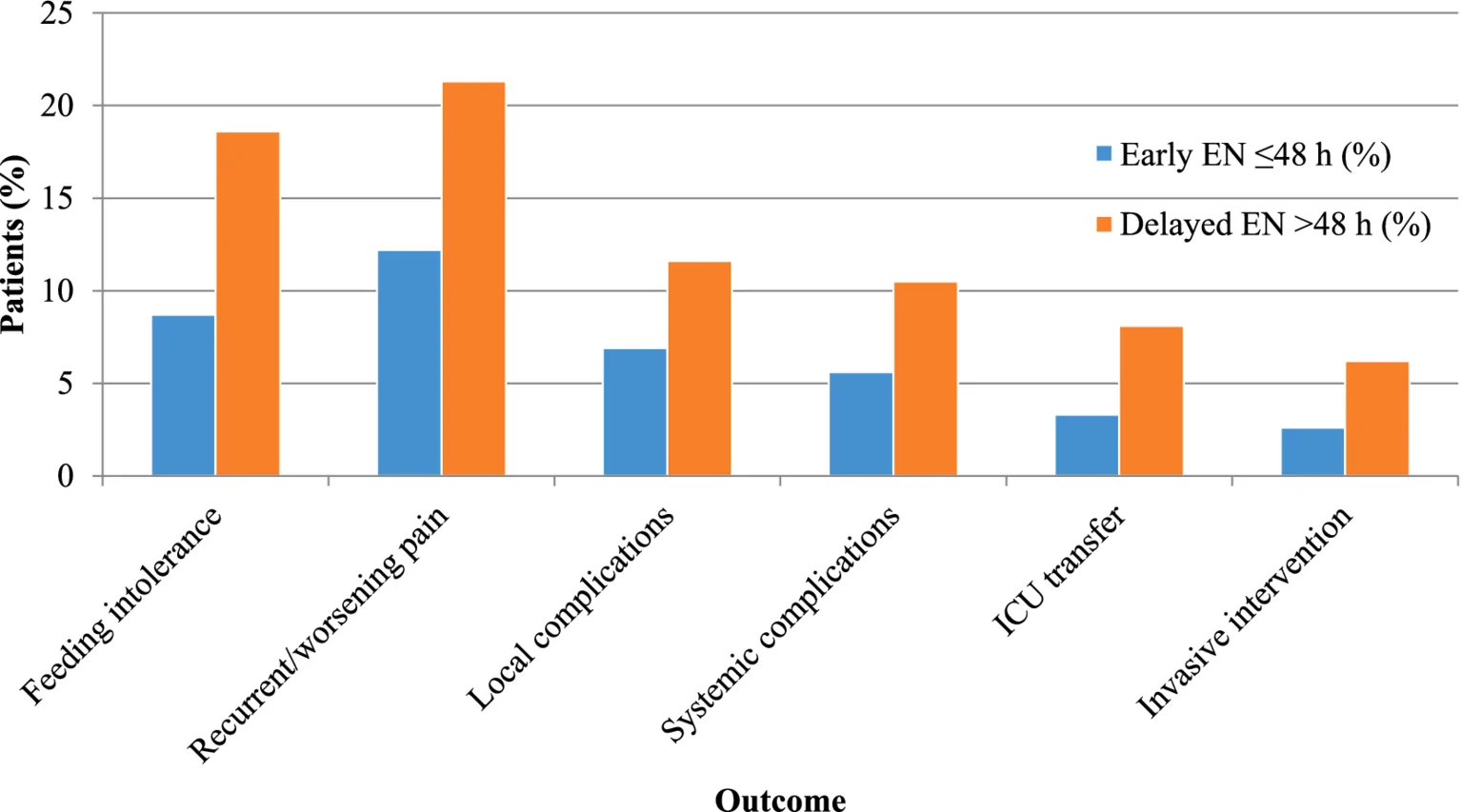
Clinical outcomes according to early and delayed enteral nutrition.

Values are presented as median (IQR), mean ± SD, or n (%). CRP, C-reactive protein; ICU, intensive care unit. Time to achievement of adequate enteral/oral intake was defined as the time from hospital admission to the first documented achievement of ≥75% of estimated daily energy and protein requirements through oral and/or enteral nutrition. The outcomes of feeding intolerance and recurrent and/or worsening abdominal pain were analyzed separately. Feeding intolerance was defined as gastrointestinal symptoms that were temporally related to oral/enteral feeding and that had an impact on nutritional intake, while recurrent/worsening abdominal pain was defined as recurrence or worsening of abdominal pain during hospitalization, regardless of its effect on feeding.

Bars represent the proportion of patients experiencing feeding intolerance, recurrent or worsening abdominal pain, local complications, systemic complications, ICU transfer, and invasive intervention in the early (≤48 h) and delayed (>48 h) enteral nutrition groups.

### 3.5 Clinical outcomes stratified by AP severity

To determine whether timing and clinical recovery differed by baseline disease severity, outcomes were also stratified into mild and moderately severe AP (Table 5). Among patients with mild AP, early EN was associated with a shorter hospital stay (5.2 vs. 6.8 days, *p* < 0.001) and lower feeding intolerance (7.6% vs. 16.5%, *p* = 0.001). A similar pattern emerged among patients with moderately severe AP, with hospital stays of 7.1 versus 9.1 days for early and delayed EN, respectively (*p* = 0.002). Although several other outcomes favored early EN in both severity strata, some comparisons did not reach statistical significance, particularly among moderately severe cases.

**Table 5:** Clinical outcomes according to enteral nutrition timing stratified by AP severity.

Outcome	Mild AP, early	Mild AP, delayed	*p* value	Moderately severe AP, early	Moderately severe AP, delayed	*p* value
Hospital stay, days	5.2 (4.1–6.7)	6.8 (5.2–8.7)	<0.001	7.1 (5.5–8.9)	9.1 (7.0–11.6)	0.002
Feeding intolerance	25 (7.6)	32 (16.5)	0.001	9 (13.8)	16 (25.0)	0.10
Recurrent pain	37 (11.3)	37 (19.1)	0.02	11 (16.9)	18 (28.1)	0.11
Local complications	15 (4.6)	19 (9.8)	0.03	12 (18.5)	11 (17.2)	0.84
ICU transfer	4 (1.2)	8 (4.1)	0.04	9 (13.8)	13 (20.3)	0.32
Median CRP, mg/L	81	103	0.01	137	165	0.09

Values are presented as median (IQR) or n (%); CRP, C-reactive protein; ICU, intensive care unit.

### 3.6 Factors associated with delayed enteral nutrition

A single multivariable logistic regression model was fitted to identify factors independently associated with delayed EN initiation (Table 6). The nine predictors shown in Table 6 were entered simultaneously into the model. After adjustment for demographic, nutritional, clinical, and biochemical characteristics, moderately severe AP was associated with higher odds of delayed EN (adjusted OR 1.72, 95% CI 1.17–2.54; *p* = 0.006). Nausea/vomiting showed the strongest clinical association (adjusted OR 2.08, 95% CI 1.45–2.98; *p* < 0.001). Higher CRP, NRS-2002 ≥ 3, and hypoalbuminemia were also independently associated with delayed nutritional initiation.

**Table 6:** Independent factors associated with delayed initiation of enteral nutrition.

Predictor	Adjusted OR	95% CI	p value
Moderately severe AP	1.72	1.17–2.54	0.006
Nausea/vomiting	2.08	1.45–2.98	<0.001
CRP, per 10-mg/L increase	1.06	1.03–1.09	<0.001
Symptom duration >24 h	1.48	1.02–2.15	0.04
NRS-2002 ≥ 3	1.61	1.08–2.40	0.02
Hypoalbuminemia	1.76	1.18–2.63	0.005
BMI, per 1 kg/m^2^ increase	1.03	0.99–1.08	0.14
Biliary etiology	0.94	0.67–1.33	0.73
Diabetes mellitus	1.19	0.78–1.82	0.42

All nine predictors shown in the table were entered simultaneously into a single multivariable logistic regression model estimating factors independently associated with delayed EN initiation. OR, odds ratio; CI, confidence interval; CRP, C-reactive protein; NRS-2002, Nutritional Risk Screening 2002.

### 3.7 Independent association between early EN and clinical outcomes

After adjustment for the prespecified covariates of age, sex, BMI, AP severity, AP etiology, symptom duration, diabetes mellitus, baseline CRP, serum albumin, serum creatinine, NRS-2002 score, nausea/vomiting, early EN remained independently associated with improved clinical recovery (Table 7). Because in-hospital mortality, invasive intervention, and ICU transfer were relatively uncommon outcomes, the corresponding adjusted estimates were considered exploratory. Given the limited number of events relative to the number of covariates included in the models, these estimates may be susceptible to overfitting and instability and should therefore be interpreted cautiously. Several secondary clinical outcomes were examined without formal adjustment for multiple comparisons, these outcome-specific associations should be considered exploratory. In particular, findings with *p*-values close to the conventional 0.05 threshold should be interpreted cautiously and viewed as hypothesis-generating rather than confirmatory. Early EN was associated with a 1.21-day shorter hospital stay (95% CI − 1.58 to −0.84; *p* < 0.001). The odds of feeding intolerance were approximately halved (adjusted OR 0.52, 95% CI 0.32–0.83; *p* = 0.006), while lower odds were also observed for recurrent/worsening abdominal pain, systemic complications, and ICU transfer. Associations with local complications, invasive intervention, readmission, and mortality were not statistically significant. Figure 3 presents the adjusted associations between early EN and individual clinical outcomes.

**Table 7:** Adjusted associations between early EN and clinical outcomes.

Outcome	Adjusted effect estimate	95% CI	*p* value
Hospital stay, days	−1.21 days	−1.58 to −0.84	<0.001
Feeding intolerance	OR 0.52	0.32–0.83	0.006
Recurrent/worsening pain	OR 0.63	0.41–0.96	0.03
Local complications	OR 0.69	0.43–1.12	0.13
Systemic complications	OR 0.58	0.33–0.99	0.047
ICU transfer	OR 0.47	0.23–0.95	0.035
Invasive intervention	OR 0.51	0.23–1.13	0.10
30-day readmission	OR 0.71	0.39–1.30	0.27
In-hospital mortality	OR 0.63	0.15–2.67	0.53

**Figure 3:**
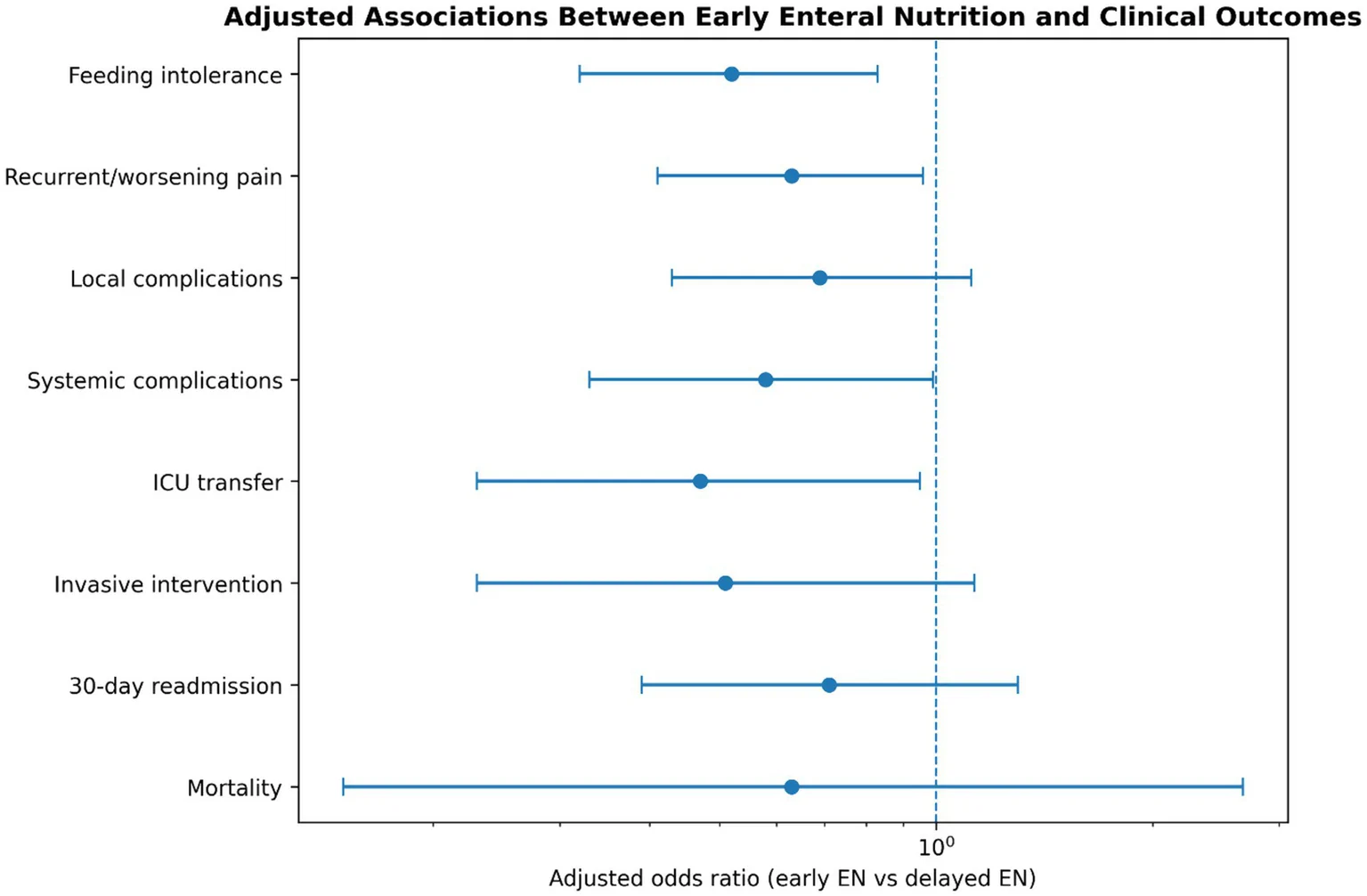
Adjusted associations between early EN and clinical outcomes.

Adjusted models included age, sex, BMI, AP severity, AP etiology, symptom duration, diabetes mellitus, baseline CRP, serum albumin, serum creatinine, NRS-2002 score, and nausea/vomiting. Because of the relatively low number of events, estimates for ICU transfer, invasive intervention, and in-hospital mortality were exploratory and should be interpreted cautiously. Because multiple secondary outcomes were assessed without formal multiplicity adjustment, these outcome-specific estimates were considered exploratory. OR, odds ratio; CI, confidence interval.

Forest plot showing adjusted odds ratios and 95% confidence intervals for clinical outcomes associated with early EN (≤48 h) compared with delayed EN (>48 h). An odds ratio below 1.0 indicates lower odds of the respective outcome in the early-nutrition group. Models were adjusted for age, sex, BMI, AP severity, AP etiology, symptom duration, diabetes mellitus, baseline CRP, serum albumin, serum creatinine, NRS-2002 score, and nausea/vomiting. Estimates for ICU transfer, invasive intervention, and in-hospital mortality were considered exploratory because of low event counts.

### 3.8 Sensitivity analysis using time to enteral nutrition as a continuous exposure

A sensitivity analysis was performed by modeling time to EN initiation as a continuous exposure rather than using the 48-h dichotomous cutoff (Table 8). Each additional 12-h delay in nutritional initiation was associated with a 0.28-day increase in hospital stay (95% CI 0.17–0.39; *p* < 0.001). Similarly, each 12-h delay was associated with increased odds of feeding intolerance (OR 1.13, 95% CI 1.06–1.21; *p* < 0.001), recurrent abdominal pain (OR 1.09, 95% CI 1.02–1.17; *p* = 0.01), and ICU transfer (OR 1.10, 95% CI 1.01–1.21; *p* = 0.03). Figure 4 illustrates the relationship between time to nutrition initiation and hospital length of stay using individual patient-level observations. Each point represents one patient, with no binning or aggregation; the fitted regression line and its 95% confidence interval illustrate the overall direction and uncertainty of the association.

**Table 8:** Sensitivity analysis of clinical outcomes according to each 12-h delay in enteral nutrition initiation.

Outcome	Adjusted effect per 12-h delay	95% CI	*p* value
Hospital stay	+0.28 days	0.17–0.39	<0.001
Feeding intolerance	OR 1.13	1.06–1.21	<0.001
Recurrent pain	OR 1.09	1.02–1.17	0.01
ICU transfer	OR 1.10	1.01–1.21	0.03
Systemic complications	OR 1.08	1.00–1.17	0.049

**Figure 4:**
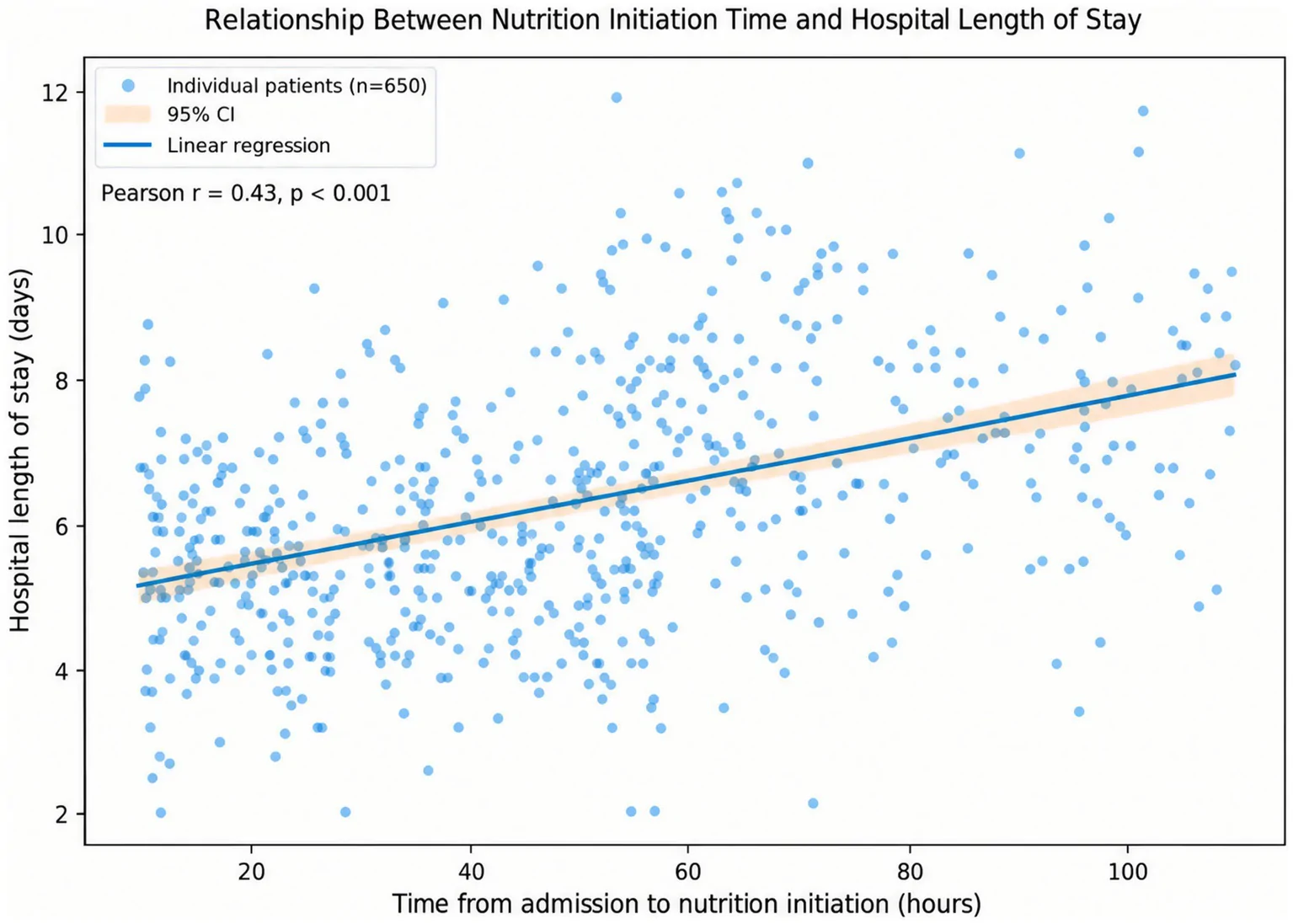
Relationship between time to EN initiation and length of hospital stay.

The sensitivity models were adjusted for age, sex, BMI, AP severity, AP etiology, symptom duration, diabetes mellitus, baseline CRP, serum albumin, serum creatinine, NRS-2002 score, and nausea/vomiting. Effect estimates represent the adjusted change in outcome associated with each additional 12-h delay in nutritional initiation. OR; odds ratio, CI; confidence interval.

Scatter plot showing individual patient-level observations for the relationship between time from hospital admission to initiation of EN and hospital length of stay. Each point represents one of the 650 patients in the analytic cohort, and no binning or aggregation was performed. The fitted line represents the linear regression relationship, and the shaded region represents the corresponding 95% confidence interval.

## 4 Discussion

In this cohort, patients who received delayed EN differed systematically from those who received early EN, with worse disease severity, more systemic derangement, and greater symptom burden, suggesting that the differences in outcomes reported below may reflect disease severity rather than feeding time. Hypertriglyceridemia was the second most common etiology in this cohort (18.6%), similar to findings from Pothoulakis et al. (11) and Olesen et al. (12), who also identified it as an important and under-recognized cause of AP internationally. The delayed-EN group also had significantly more moderate-to-severe disease (24.8% vs. 16.6%), higher blood pressure, heart rate, and temperature, longer symptom duration, and was significantly more likely to have moderate-to-severe abdominal pain, nausea/vomiting, and abdominal distention, which parallels the findings of Pothoulakis et al. (13) that gastrointestinal symptoms are common in AP and are a reason for the development of feeding intolerance, but not a separate risk factor.

The delayed-EN group also had a poorer nutritional and inflammatory status, indicated by higher NRS-2002 scores, higher nutritional risk and lower serum albumin. Hypoalbuminemia is also common in AP, as reported by Ocskay et al. (14), who described it as an independent risk factor for a more severe and more delayed EN patient profile; however, in this study, we did not examine hypoalbuminemia as an independent predictor of outcome. In line with this, Chen et al. (15) showed that the NRS-2002 score was an effective tool for identifying patients at higher nutritional risk in severe AP; similarly, in our delayed-EN group, the NRS-2002 score was higher. In accordance with the findings of Walkowska et al. (7), who reported inflammatory, renal, metabolic and electrolyte abnormalities as important elements of clinical assessment in AP, delayed EN was also correlated with higher levels of CRP, leukocytes, BUN, creatinine and glucose, and lower levels of lymphocytes, indicating a greater degree of systemic inflammation, physiological stress and metabolic disturbance at admission.

This study also showed a clear difference in the nutritional timing between groups (median time for EN initiation: 31 h vs. 70 h) and almost half of early-fed patients began feeding within 24 h. Our results complement the Liang et al. (16) meta-analysis of early EN strategies versus withheld or delayed EN nd their downstream consequences, which found that earlier feeding was associated with better clinical outcomes, but which mainly compared broad feeding strategies (early EN versus withheld or delayed EN) and their downstream outcomes rather than examined the specific hour-by-hour thresholds for initiating enteral nutrition (EN) as evaluated here; the present finding extends this literature by showing that the actual timing of the initiation of enteral nutrition (EN) itself, both as a dichotomy at 48 h and as a continuous exposure in the sensitivity analyses, is independently associated with outcomes within a cohort already receiving enteral feeding. Similarly, Horibe et al. (17) reported that immediate oral feeding was not harmful and was effective in mild-AP, consistent with the high percentage of early-feeding patients in our mild-AP group (92.3%). Higher rates of persistent nausea/vomiting, ileus, and abdominal pain among delayed EN patients compared to early EN patients are reflected by the higher rates of feeding interruptions, nutritional supplementation, and tube-based EN, which is consistent with Lin et al. (18), who noted gastrointestinal dysfunction and feeding intolerance as barriers to enteral nutrition in moderately severe to severe AP.

In this study, early EN was significantly associated with clinical outcomes such as shorter hospital stays, earlier pain resolution, earlier reach of sufficient EN intake, and fewer feeding intolerances and recurrent pain. These associations are consistent with the meta-analysis by Yao et al. (19), which revealed that there was no evidence of increased adverse outcomes with immediate/early EN after AP and reported better recovery, and with Guo et al. (20), who reported that immediate feeding of EN was safe and associated with faster recovery in mild AP, similar to our early-EN, predominantly mild-AP group. Early EN was also associated with decreased peak CRP, increased discharge albumin, and more CRP reduction during discharge (toward better inflammatory and nutritional recovery), which was consistent with the results of a large Japanese AP database reported by Kusanaga et al. (21); lower rates of local and systemic complications, ICU transfer, and invasive intervention (directionally consistent with better recovery of inflammatory and nutritional status); and lower rates of local and systemic complications, ICU transfer, and invasive intervention (directionally consistent with better recovery of inflammatory and nutritional status), were observed.

The association between early EN and favorable outcomes was similar in the mild and moderately severe AP groups but varied in strength and consistency across subgroups. Among the mild-AP subgroup, early oral/enteral feeding correlated with fewer local complications, less recurrent pain, shorter hospital stays, less feeding intolerance, less ICU transfer and lower median CRP, a trend indicative of a favorable safety and outcome profile for early EN in less severe disease. This aligns with the PADI trial, which showed that immediate oral refeeding was safe even in mild to moderate AP without compromising clinical recovery (22). Similarly, moderate-to-severe AP has shown positive results when feeding is based on hunger, and early initiation of solid food (within 24 h) improved outcomes without increasing feeding-related problems (23, 24). These studies help demonstrate that hunger-based feeding advances may be successful across a range of AP severities.

When the actual direction was examined, there were no significant differences in local complications between the two groups among patients with moderately severe AP, with a slightly higher proportion in the early-EN group (18.5% vs. 17.2%, *p* = 0.84). Interpret this small, non-significant directional reversal with caution, especially given the smaller subgroup size and limited number of events.

In multivariable analysis, some clinical and nutritional factors were independently associated with delayed EN initiation. Disease severity and the presence of nausea/vomiting were the two most important independent risk factors – moderate AP was associated with a 72% increased risk of delayed EN, and nausea/vomiting was the strongest independent risk factor, showing that disease severity and ongoing gastrointestinal symptoms are the two biggest inhibitors to early nutritional progress. In a similar study by Nammour et al. (25), oral feeding was started earlier than commonly suggested and should not be delayed simply because of perceived symptom severity. The independent associations of NRS-2002 ≥ 3, hypoalbuminemia, and delayed EN indicate that there are higher barriers to providing early nutritional support for patients at higher nutritional risk, consistent with the ESPEN guideline recommendation to tailor nutritional assessment and nutritional support (EN) initiation based on the disease severity and the patient’s tolerance (8). Greater inflammatory burden (defined by elevated CRP), longer illness duration (>24 h), and delayed EN further confirm an independent association of these variables, as Kazmi et al. (26) previously described, noting that CRP and albumin abnormalities are clinically important markers of AP severity.

Early EN was independently associated with better outcomes after adjusting for demographic, disease-severity, nutritional, and biochemical factors, including a 1.21-day reduction in hospital stay and a reduced risk of feeding intolerance, recurrent/worsening pain, systemic complications, and ICU transfer. Chatterjee et al. (27) confirmed these findings, reporting good outcomes with nutritional interventions started within 48 h in predicted severe AP, and Li and Zhang (28) associated earlier nutritional intervention with shorter hospital stays in severe AP. The lack of significant differences in local complications, invasive intervention, 30-day readmission, and mortality may reflect that these events are uncommon in this cohort and may be more strongly associated with short-term recovery and tolerance of early EN than with these fewer common outcomes. Kaur et al. (29) also support enteral nutrition over feeding restriction or parenteral feeding for AP.

The sensitivity analysis confirmed these results, finding that there was a progressive relationship between nutritional delay and clinical outcomes (not a threshold effect), with an additional 12 h delay resulting in a 0.28-day extension of hospital stay and a sequential increase in risk of feeding intolerance, recurrent pain, transfer to the intensive care unit, and systemic complications. This dose–response relationship aligns with the systematic review by Liu et al. (30), which associated enteral feeding in AP with improved clinical outcomes and demonstrated the benefits of avoiding unnecessary feeding delays, and with Malali et al. (31), who found that delayed EN was associated with less favorable clinical recovery and recommended individualizing timing for feeding according to tolerance rather than prolonged fasting. Li et al. (32) provided additional context for the observed increase in feeding intolerance with increasing nutritional delay by examining factors associated with enteral nutrition intolerance.

### 4.1 Strengths of the study

This study has several important strengths. First, it included a relatively large number of patients with AP (650), allowing assessment of the relationship between time to enteral nutrition (EN) and clinical outcomes. Second, patients were ranked using a well-established 48-h window for early vs. delayed EN, allowing clinical assessment of nutritional timing. Third, the study assessed a wide array of outcomes, including hospital stay, pain resolution, adequate intake, feeding intolerance, inflammatory response, complications, transfer to an intensive care unit, invasive intervention, readmission, and mortality. Fourth, the analysis included severity-stratified comparisons and multivariable adjustment, strengthening interpretation of the observed associations by accounting for important demographic, nutritional, clinical, and biochemical factors. Finally, the continuous-exposure sensitivity analysis confirmed that greater delay in EN was increasingly correlated with worse outcomes, providing further evidence for the robustness of the main results, which were based on dichotomising EN after 48 h.

### 4.2 Limitations of the study

Consider the following when interpreting these results. First, the study is observational, so it cannot establish a definitive causal relationship between early EN and improved outcomes. Adjustment for other variables was made, but residual confounding and unmeasured confounding may exist. Second, delayed EN may partly reflect increased disease severity and/or gastrointestinal symptoms (nausea, vomiting, ileus, and persistent abdominal pain), which may also lead to poorer outcomes. Third, the study was conducted in a specific clinical group, so the results may be less generalizable to other hospitals, healthcare systems, or populations. Fourth, although several clinically important variables were adjusted for, other factors, such as detailed fluid management, use of analgesics, changes in nutrition protocols and physician-specific decisions about feeding, may not have been captured. Lastly, the number of events for in-hospital death, invasive intervention, and ICU transfer was relatively small compared with the number of covariates used in the adjusted models. These estimates may therefore suffer from overfitting and statistical instability and should be viewed as exploratory, not definitive.

### 4.3 Future recommendations

Future studies should involve larger, prospective, multicenter studies and RCTs to further clarify the benefit, or lack thereof, of early EN in AP. Studies should also examine the effects of optimal timing by severity, nutritional risk, gastrointestinal tolerance, and underlying etiology. Standardizing feeding protocols, including the choice between oral and tube EN, diet composition, and management of feeding intolerance, would improve comparability across studies. Future studies should also assess long-term effects (such as recovery of nutritional status, recurrent pancreatitis, quality of life, and nutritional status after discharge). In addition to time-to-feeding as a continuous exposure, further research using this method could help determine a clinically useful window for nutrition initiation, rather than relying on an arbitrary time.

## 5 Conclusion

Enteral nutrition within 48 h was consistently associated with more favorable clinical outcomes in this group of 650 patients with AP. Early EN was associated with shorter hospital length of stay, earlier pain resolution and adequate intake, reduced feeding intolerance, earlier decreases in CRP levels, improved albumin levels at discharge, and fewer local and systemic complications, ICU transfers, and invasive interventions. These associations persisted after adjustment for key demographic, nutritional, clinical, and biochemical factors. Importantly, the sensitivity analysis showed a progressive increase in adverse outcomes with each additional 12-h delay in nutrition, suggesting a time-dependent relationship between nutritional delay and adverse outcomes. In conclusion, these results support using enteral nutrition as a key part of supportive treatment in AP, and larger prospective studies should be conducted to support causality and determine optimal timing and nutritional approaches for different patient subpopulations.

## Data Availability

The raw data supporting the conclusions of this article will be made available by the authors, without undue reservation.
